# Locomotion and Topographical Working Memory in Children With Myelomeningocele and Arthrogryposis Multiplex Congenita

**DOI:** 10.3389/fpsyt.2021.729859

**Published:** 2021-11-12

**Authors:** Åsa Bartonek, Cecilia Guariglia, Laura Piccardi

**Affiliations:** ^1^Neuropaediatric Unit, Department of Women's and Children's Health, Karolinska Institutet, Stockholm, Sweden; ^2^Department of Psychology, University Sapienza of Rome, Rome, Italy; ^3^Cognitive and Motor Rehabilitation and Neuroimaging Unit, IRCCS Fondazione Santa Lucia, Rome, Italy

**Keywords:** disability, mobility, motor development, navigation, walking, spatial cognition

## Abstract

**Background:** In children with myelomeningocele (MMC) and arthrogryposis multiplex congenital (AMC), adequate rehabilitation measures are accessible with the goal of attaining the utmost motor development. However, there is a lack of knowledge as to how children develop navigation utilizing their locomotion abilities. The aim of the present study was to explore topographic working memory in children with MMC and AMC.

**Methods:** For this purpose, we assessed 41 children with MMC and AMC, assigned an ambulation group, and 120 typical developing (TD) children, with mean ages of 11.9, 10.6, and 9.9 years, respectively. All groups performed a topographic working memory test while moving in a walking space and a visuospatial working memory test in a reaching space. Children with MMC and AMC also performed a test to measure their ability to reason on visuospatial material, Raven's Coloured Progressive Matrices.

**Results:** The topographic working memory span was shorter in the MMC group than in the TD group. In general, all ambulation groups had a shorter topographic working memory span than the TD group. The visuospatial working memory span was shorter in the non-ambulation group than in the TD group. Scores from the visuospatial reasoning test were lower in the non-ambulation group than in the community ambulation group.

**Conclusions:** Even though a higher cognitive score was found in the community ambulation group than in the non-ambulation group, topographic working memory was affected similarly in both groups. Including children who develop community ambulation in therapy programs containing aspects of navigation may gain even children with low levels of MMC and AMC. These results evidenced the importance of motor development and navigational experience gained through direct exploration of the environment on topographic memory.

## Introduction

In children with myelomeningocele (MMC) and arthrogryposis multiplex congenita (AMC), various modes of locomotion from independent walking to wheelchairs are present, and adequate rehabilitation measures are accessible with the goal of attaining the utmost motor development (1, 2). To achieve locomotion that can be expected with respect to each child's capability, various interventions are offered by the medical team. There is, however, a lack of knowledge as to how children develop navigation utilizing their locomotion abilities.

MMC, a subgroup of *spina bifida*, is a condition characterized by the failure of the lumbosacral spinal neural tube to close during embryonic development, resulting in neurological deficits that vary with the level of the lesion (3). Apart from primary paresis, covering sacral to thoracic muscle paresis levels, consequences of hydrocephalus and secondary damage to the spinal cord may hinder a child's walking function (4). In children with MMC, a characteristic gait pattern has been identified (5), and motor development and orthotic treatment during childhood with respect to each child's expected ambulatory level has been recommended (1).

AMC can be of extrinsic aetiology, resulting from intrauterine congenital abnormalities such as foetal akinesia, or of intrinsic aetiology, due to abnormalities in the central nervous system (6). Children with AMC present multiple problems that necessitate multidisciplinary management in the form of intensive physiotherapy, bracing and surgery (7), with orthotic support ensuring that even children with severe muscle weakness and joint contractures achieve walking (8). In children with amyoplasia, the most common form of AMC, attention should be paid to developing muscle strength and minimizing periods of immobilization to stimulate active movement early in life (2).

Locomotion is related to navigation in the sense that children's memory of how a space is mapped out is linked to how they move through that space (9). In children with typical development (TD), the use of body movement information contributes to spatial updating at the end of the first year of life (10). In children between 4 and 6 years of age, those who actively directed their own route in spatial memory tasks performed better than children who passively experienced the same route (11). In infants with MMC with low sacral lesions, a highly significant effect of delayed locomotor experience was found, with the infants tending to look at the experimenter's face and not towards the general region of the head turn, point, and gaze (12). Compared to children without disability, children with *spina bifida* and hydrocephalus were more dependent on landmarks in the environment to find their way (13). When comparing wayfinding choices in teenagers with physical disabilities, comprising individuals with MMC and AMC, those who experienced limited mobility early in development performed poorer than those whose mobility deteriorated with age (14). Since infants and children with limited exploration are at risk for global developmental impairments, early physiotherapy interventions targeting exploratory behaviours have been recommended to minimize future delays in children with special needs (15).

Spatial development builds on important beginnings in the neural systems of newborns but changes in experience-expectant ways with motor development. Over the first 2 years of human life, babies rapidly acquire motor skills that give them increasingly independent and wide-ranging access to the environment (16). The definition of spatial cognition comprises mental representation of near, peri-personal and reaching space, as well as that of far, extra-personal and navigational space, which are processed by partially separated neurocognitive systems (17, 18), the latter developing later during childhood (19). Spatial cognition includes the ability to memorize the positions and locations of objects in the environment, spatial planning by using memorized information for acting or moving in space, and awareness of spatial features. Topographic working memory, within spatial cognition, enables the encoding and maintaining online sequences of environmental cues that are significant during navigation and orienting in familiar environments (20, 21).

In children with MMC and AMC, planning for expected locomotion ability is practised from an early age with physiotherapy and orthotic management. The main aim of the present study was to explore topographic working memory in children who had access to rehabilitation and care from childhood with the goal of achieving utmost motor development. Since it may be expected that movement experiences in the environment are affected in children with motor disabilities originating from birth, our hypothesis was that children with MMC and AMC would present worse topographic working memory than children with TD development. In children with MMC, higher visuospatial function was found in children with independent mobility than in children who required assistance for mobility, possibly associated with difficulty obtaining a comprehensive picture of the surroundings interfering with their mobility (22). *A second purpose was therefore to investigate the participants' visuospatial working memory in the reaching space since it is well-known that the efficiency of visuospatial memory predicts navigational ability to the extent that good navigators have a greater capacity for visuospatial working memory* [e.g., (23–25)].

## Materials and Methods

### Participants

Eighty-nine children with motor disabilities were consecutively included in a project on topographic working memory between January 2014 and December 2016. In the present *case-control study*, we report 41 children (22 males, 19 females), of whom 31 had MMC, with a mean age of 11.9 (S. D. of 3.2) (19 with shunted hydrocephalus, 61%) and 10 with AMC, mean age of 10.6 (S. D. of 3.1). All participants were patients at Karolinska University Hospital, Stockholm. A total of 120 TD children (57 males, 63 females), mean age 9.9 (S. D. of 3.1), constituted a control group recruited among siblings of inpatients and through advertisements in the hospital. The study was approved by the Regional Ethical Review Board in Stockholm. Parents gave informed written consent, and children provided verbal assent before taking part in the study. All children had to understand the instructions necessary to perform the tests.

### Ambulation Groups

The participants were assigned to a group of ambulations, i.e., community ambulation walking outdoors, household ambulation walking indoors and using wheelchair outdoors, or to a non-ambulation group using a wheelchair for all transfers (26, 27).

### Visuospatial and Topographic Working Memory

All participants performed two working memory tests, a visuospatial memory test (Corsi block-tapping Test, CBT) (28) in the reaching space and a topographic working memory test (Walking Corsi Test, WalCT) (29) in randomized order.

**Figure 1:**
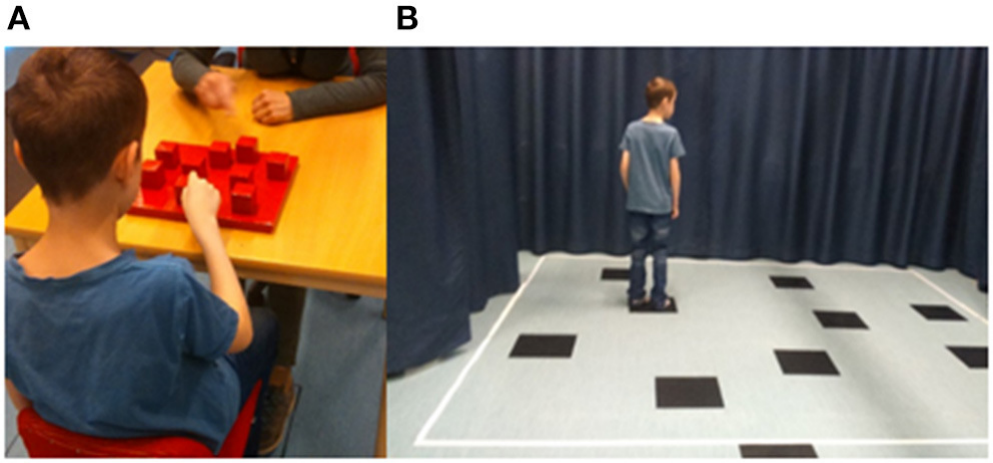
**(A)** Apparatus used to administer the Corsi block-tapping test (CBT). The CBT consists of nine wooden blocks (4.5 × 4.5 cm) fixed on a baseboard (30 × 25 cm). Numbers are only present on the examiner's side, not visible to the child. **(B)** The walking Corsi test (WalCT) is a larger version of the CBT (300 × 250 cm) consisting of nine squares placed on the floor in identical positions as in the CBT.

The CBT consists of nine wooden blocks (4.5 × 4.5 cm) fixed on a baseboard (30 × 25 cm) in a scattered array and numbered on the examiner's side for ease of identification, not visible to the participant (Figure 1A). The examiner taps a number of blocks at a rate of one block per 2 s, lifting the hand straight up before moving it to the next block, after which the participant taps the block sequence in the same order. Starting from a two-block sequence, the examiner gradually increases the length of block numbers to a maximum of nine. The participants were tested individually in a quiet room seated on a height-adjustable chair in front of the CBT baseboard facing the experimenter.

WalCT assesses topographic memory in a vista space, namely, the space that the individual can see from a single location or with only little exploratory movements, such as single rooms or town squares (29, 30). It is a larger version of the CBT (300 × 250 cm) consisting of nine squares placed on the floor in identical positions as in the CBT. The WalCT area was set up in a part of a room visualized by straps on the floor and encircled by textile curtains in front and at the sides of the WalCT area (Figure 1B). Beginning with a two-square sequence, the examiner illustrates the sequence by walking on the squares and stopping on each of them for 2 s, gradually increasing the length of square sequences. In both CBT and WalCT, five trials are presented, of which three trials must be correctly performed to continue with the next higher sequence. The score is equivalent to the longest sequence (span) repeated correctly by the participant.

All participants in the non-ambulation group performed the WalCT in their habitually used manual wheelchair. The participant was instructed to stop on the square in a manner that the square was still observable by her/himself, which was possible through the footplate of the wheelchair. To check for influence on working memory (31), WalCT was also tested in 11 children in the non-ambulation group when using a laser pointer after the examiner illustrated the sequence by walking on the squares. A paired *T*-test did not reveal any difference in spans (*p* = 0.504) between pointing the sequence from a static position (mean 4.42, S. D 1.24) and stopping on the squares with the wheelchair (mean 4.25, SD 0.96).

### Visuospatial Reasoning Test

A visuospatial reasoning test, Raven's Coloured Progressive Matrices (CPM) (32), was performed in children with MMC and AMC. The CPM consists of 36 items and assesses non-verbal abilities by measuring clear-thinking ability for young children from 5 years of age. The CPM produces a single raw score that can be converted to a percentile based on normative data.

### Statistical Analysis

Descriptive data are presented as the mean and standard deviation. Analysis of variance (ANOVA) with *post-hoc* analysis and Fisher's least significant difference (LSD) was used to analyse sex and age between groups and to analyse differences in WalCT and CBT spans with age as covariates between the TD and MMC/AMC groups and between the TD and AMB groups, as well as the CPM total score between the MMC and AMC groups and between the ambulation groups. A paired *T*-test was used to compare WalCT and CBT spans in the TD, MMC and AMC groups and between the TD and ambulation groups. A chi-square-test was used to calculate hydrocephalus in the MMC group. The alpha level chosen for considering a statistical test as significant was *p* <0.05. All statistics were conducted using IBM SPSS software version 26.

## Results

There was no difference in sex between the MMC, AMC and TD groups (*p* = 0.713) or between the ambulation groups (*p* = 0.493). Among the MMC participants, shunted hydrocephalus was significantly more present in the non-ambulation group (13/14) than in the household ambulation group (3/9) and the community ambulation group (3/8) (*p* = 0.005).

Children in the TD group were significantly younger than those in the MMC group, *F*_(2.158)_ = 5.287; *p* = 0.006; *partial g2* = 0.063 (Table 1) and significantly younger than those in the Na group, *F*_(3, 157)_ = 3.697; *p* = 0.013; *partial g2* = 0.066 (Table 2).

**Table 1:** Demographics, mean, standard deviations (SD), age and spans of WalCT and CBT, and distribution of ambulation with respect to participants with MMC and AMC.

	**TD *n* = 120**	**Ca*n* = 16**	**Ha *n* = 11**	**Na*n* = 14**	***p***	**TD–Ca**	**TD–Ha**	**TD—Na**	**Ca–Ha**	**Ca–Na**	**Ha–Na**
DiagnosisMMC/AMC	-	8/8	9/2	14/0	*-*						
Sex f/m	63/57	6/10	6/5	7/7	0.721						
Age (years) mean (SD) (min-max)	9.89 (3.11) (5–16)	11.31 (3.44) (5–17)	10.95 (2.85) (5–17)	12.53 (3.4) (5–17)	**0.013**	**0.013**	0.287	**0.004**	0.773	0.292	0.217
WalCT span mean (SD)	4.22 (1.4)	4.06 (1.4)	3.82 (0.9)	4.07 (0.9)	**0.002**	**0.037**	**0.028**	**0.002**	0.728	0.356	0.616
CBT span mean (SD)	4.79 (0.94)	4.86 (1.2)	4.82 (1.07)	4.64 (1.01)	**0.032**	0.358	0.485	**0.004**	0.968	0.102	0.127
Raven CPM Total score	-	28.53 (6.43)	26.45 (7.07)	23.79 (8.27)	**0.033**	-	-	-	0.518	**0.011**	0.077

*MMC, myelomeningocele; AMC, Artrogryphosis multiplex congenita; Ca, community ambulation; Ha, household ambulation; Na, non-ambulation; TD, typical developing children*.

**Table 2:** Age and spans of walking Corsi test (WalCT) and Corsi block test (CBT) and Raven's CPM total in groups of typically developing children (TD) and children in various ambulation groups.

	**TD *n* = 120**	**Ca *n* = 16**	**Ha *n* = 11**	**Na *n* =14**	***P***	**TD–Ca**	**TD–Ha**	**TD–Na**	**Ca–Ha**	**Ca–Na**	**Ha–Na**
Age (mean, SD) min-max yrs	9.89 (3.11) (5–16)	11.31 (3.44) (5–17)	10.95 (2.85) (5–17)	12.53 (3.4) (5–17)	**0.013**	**0.013**	0.287	**0.004**	0.773	0.292	0.217
WalCT span (mean, SD)	4.22 (1.4)	4.06 (1.4)	3.82 (0.9)	4.07 (0.9)	**0.002**	**0.037**	**0.028**	**0.002**	0.728	0.356	0.616
CBT span (mean, SD)	4.79 (0.94)	4.86 (1.2)	4.82 (1.07)	4.64 (1.01)	**0.032**	0.358	0.485	**0.004**	0.968	0.102	0.127
Raven CPM Total score	-	28.53 (6.43)	26.45 (7.07)	23.79 (8.27)	**0.033**	-	-	-	0.518	**0.011**	0.077

*Ca, community ambulation; Ha, household ambulation; Na, non-ambulation. Statistically significant differences (P <0.005) in bold. TD, typical developing children*.

### WalCT and CBT in Groups of MMC and AMC

**Figure 2:**
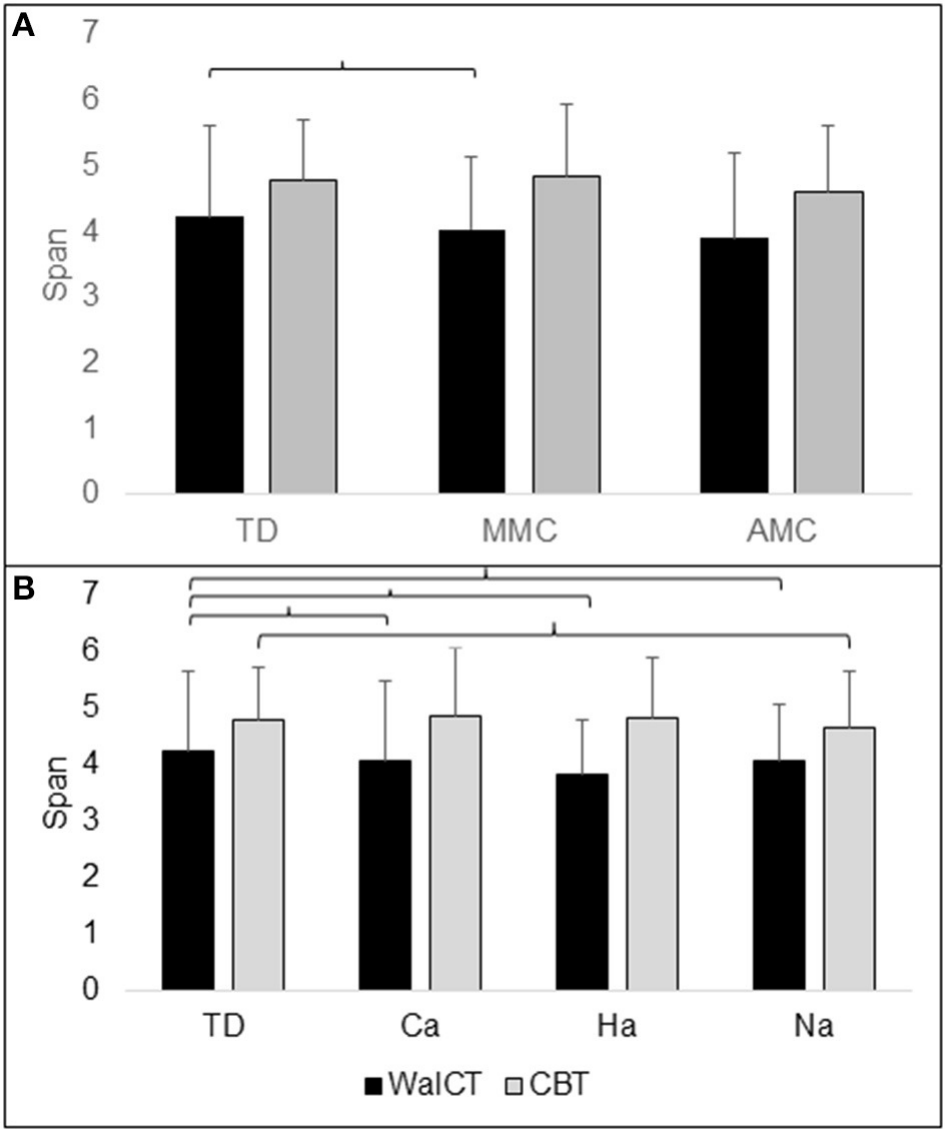
Illustration of topographical (WalCT) and visuospatial (CBT) working memory in **(A)** groups of TD (typical developing children), MMC (myelomeningocele) and AMC (artrogryphosis multiplex congenita) and **(B)** groups of Ca (community ambulation), Ha (household ambulation) and Na (non-ambulation). The number of spans with standard deviation (SD) is shown on the y-axis. Parentheses indicate significant difference.

Between the TD, MMC, and AMC groups analysed with age as a covariate, WalCT span differed significantly, *F*_(2, 157)_ = 5.579; *p* <0.001*; partial g2* = 0.088. *Post-hoc* LSD tests revealed that the WalCT score was lower in the MMC group than in the TD group. CBT span did not differ between the TD, MMC, and AMC groups, as analysed with age as a covariate, *F*_(2, 767)_ = 2.579; *p* = 0.066; *partial g2* = 0.034 (Table 1; Figure 2A).

### WalCT and CBT in Ambulation Groups

WalCT span differed significantly between the TD and ambulation groups with age as a covariate, *F*_(3, 156)_ = 5.179; *p* <0.002; *partial g2* = 0.091. *Post-hoc* LSD tests revealed that the WalCT score was lower in all ambulation groups than in the TD group. CBT span differed significantly between ambulation groups with age as a covariate, *F*_(3, 156)_ = 3.009; *p* = 0.032; *partial g2* = 0.055. A *post-hoc* LSD test revealed that the CBT score was lower in the non-ambulation group than in the TD group (Table 2; Figure 2B).

### CPM in Groups of MMC and AMC and Ambulation Groups

**Figure 3:**
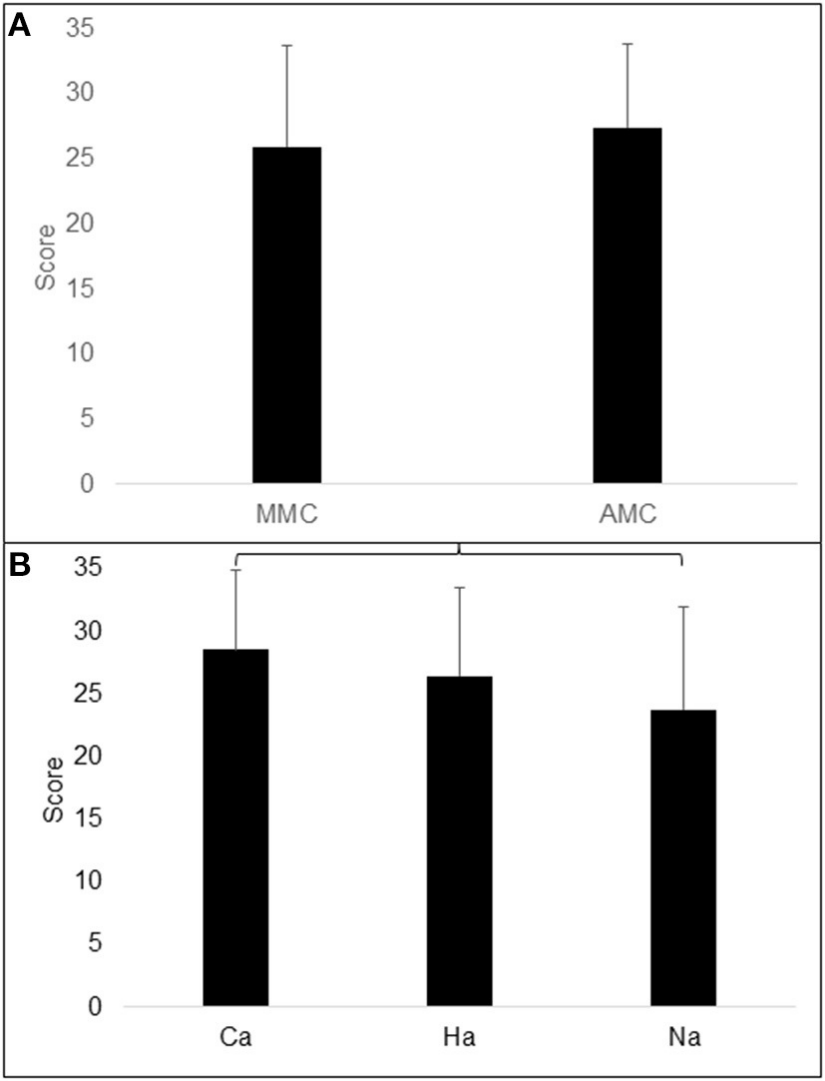
Illustration of Raven total score (CPM) in **(A)** groups of MMC (myelomeningocele) and AMC (artrogryphosis multiplex congenita) and **(B)** in groups of Ca (community ambulation), Ha (household ambulation) and Na (non-ambulation). Parentheses indicate significant difference.

With age as a covariate, no difference was found in CPM between the MMC and AMC groups, *F*_(1, 37)_ = 1.514; *p* = 0.226; *partial g2* = 0.039 (Table 1). CPM differed significantly between the ambulation groups with age as a covariate, *F*_(2, 36)_ = 3.774; *p* = 0.033; *partial g2* = 0.172. *Post-hoc* LSD tests revealed that CPM was lower in the non-ambulation group than in the community ambulation group (Table 2; Figures 3A,B).

## Discussion

The main purpose of this study was to explore topographic working memory in children with MMC and AMC when using their sedentary manner of locomotion. *The main finding was that the topographical working memory score was significantly lower in children with MMC than in the TD group when analysed according to diagnosis groups. When dividing the children according to their functional ambulation, however, all groups performed worse in topographical working memory than the TD group*.

### WalCT

In accordance with our hypothesis, both children with MMC and AMC performed more poorly on the topographic working memory test than children in the TD group. In the AMC group, the result was not statistically significant, which may be due to the lower age but also to the smaller sample size in the AMC group than in the TD group. Moreover, no differences were found in WalCT between the MMC and AMC groups. When dividing the MMC and AMC participants into community, household and non-ambulation ambulation groups, all three groups performed worse in topographic working memory than the TD group. In the community ambulation group, the MMC and AMC groups were equally represented, and both groups presented with muscle paresis, muscle weakness or joint deformities, mainly at the distal parts of the legs. The household ambulation group consisted of nine participants with MMC and two with AMC, all of whom required orthoses to stabilize knee and hip joints, enabling stable weight-bearing on the lower limbs. During the WalCT test, the participant had to turn their body on the test squares in various directions. This may have required more attention on the motor task in children in the household ambulation group with hip muscle weakness than in the community ambulation group most often presenting with calf muscle weakness using low leg orthoses, possibly influencing performance. In the non-ambulation group, there were only participants with MMC, all of whom were dependent on wheelchairs for locomotion due to extensive lower limb muscle paresis. Testing in a wheelchair could be assumed to require more time to perform the test than by walking, thus resulting in a higher cognitive load for topographic working memory. Nevertheless, when testing eleven patients in our study group with both pointing the sequence with a laser pointer from a static position and driving a wheelchair, no difference was found, which was confirmed by the results of De Nigris et al. (31), who found no difference between pointing at a WalCT sequence and performing the route by walking. We therefore do not assume time duration to have produced a crucial effect on the results. In contrast, performing the test in a wheelchair could possibly have contributed to less physical effort than performing the test in bipedal locomotion with the use of orthoses.

### CBT

In contrast to topographic working memory, no statistically significant differences were found between the TD, MMC and AMC groups in the visuospatial working memory test. All three groups reached similar scores, even though somewhat lower scores were found in the AMC group than in the MMC group may be explained by the younger age in the former group. Compliant with topographic working memory, however, there was a difference according to ambulation groups showing significantly lower visuo-spatial working memory score in the non-ambulation group, consisting of only participants with MMC, compared to the TD group. This may be in accordance with findings of visuospatial deficits in children with MMC, whereby the children who needed assistance for mobility performed more poorly in visuospatial function than those with independent walking (22).

### WalCT vs. CBT

All ambulation groups, as well as the TD group, achieved higher CBT scores than WalCT scores. This may be because of the different test situations, in which WalCT was performed while moving and turning the body in space, whereas the CBT test was accomplished in a static position while sitting. A spontaneous reflexion by a TD participant was that she had to switch direction in her brain when turning the body on the squares, which had not been necessary at the CBT test while sitting. Memorising a sequence of block tapping in a baseboard (CBT) and memorising a sequence of steps in a walked path (WalCT) thus appears to rely on different processes and strategies (29).

### CPM

To provide a measure of the participants' cognitive development, the participants with MMC and AMC were tested in their capability to reason on visuospatial material (32). CPM data were missing in one participant with MMC who, however, attended regular school and had no hydrocephalus. There was no difference in CPM score between the MMC and AMC groups. According to ambulation groups, the total CPM score declined from community and household to the non-ambulation group, differing significantly between the community and non-ambulation groups. In the non-ambulation group, consisting of only MMC participants, hydrocephalus was more frequently present than in the community and household ambulation groups. In children with MMC, some types of neurocognitive operations are described as being intact, and others are impaired, among others, in spatial orientation (33). Moreover, a higher level of spinal lesions in children with MMC and hydrocephalus has been associated with poorer neurobehavioural outcomes that determine levels of independent functioning (34), which may contribute to explaining the lower CPM score in the non-ambulation group than in the community ambulation group. Concerning the AMC participants, most of them were in the community ambulation group; thus, the findings in this study do not point to difficulties with visuospatial reasoning in children with AMC.

### Mobility Experiences

Findings from studies in children and adolescents with motor disabilities suggest that reduced route knowledge and wayfinding are most likely due to missing mobility experiences (13). Structured training at an early age has been proposed to help affected children become more efficient at finding their way in the neighbourhood (35). Furthermore, early independent exploration for the development of spatial knowledge is recommended to avoid the unfavourable effects of limited early exploratory experience to persist into the teenage years (14). In the study of Campos et al. (12), delayed onset of locomotion in children with MMC at low sacral lesions was reported to occur between 4 and 7 months, remaining in the prolonged prone position. Today, in the rehabilitation of children with MMC, also in sacral lesions, an upright position with supporting ankle orthoses is offered to be initiated as soon as the child starts crawling. From a medical viewpoint, this measure intends to avoid muscular imbalance between paretic and non-paretic muscles at an early age (1). Finding an accurate appointment with the child's intension of motor development, however, may lead to the development of attentional skills that generalize to contexts other than those involving locomotion (12).

Among children with AMC, some achieve independent walking between 1 and 2 years of age, whereas walking is delayed until 5 years of age, mostly in those with household ambulation (2). Recently, it has been reported that spatial development begins already in the neural systems of newborns and that children expand their ability to encode and combine various sources of spatial information from 3 to 10 years of age (16). However, even if active rehabilitation is accomplished at an early age with effective orthotic management in both MMC and AMC (36), it is not probable that the majority of children have the possibility to make environmental explorations as a typical developing child. In this sense, the recommendations seem useful for some youth with disabilities to take part in travel training interventions to support learning in pedestrian and public transportation navigation skills (37). In the case of locomotion, the education of team members has been recommended to provide daily opportunities for a child to be mobile among caregivers and peers rather than locomotion in isolated environments (15).

### Limitations and Strengths

A larger sample of individuals with various diagnoses with outdoor walking showed improved topographic memory compared to individuals who did not walk outdoors and to individuals who were mobile through wheelchairs (38). The same results could not be confirmed in the present study, which may partly be due to the small participant number, particularly in the AMC group, which is a rare disease with the possibility of limited patient recruitment. This can be considered a shortcoming of the study, not allowing any general assumption. Nevertheless, the findings in this study are specific to children with MMC and AMC, both groups with characteristic motor development. *Together with knowledge about specific requirements to achieve expected ambulatory goals, the findings contribute to highlighting the importance of the utmost development of topographical working memory in these patient groups*. Since spatial development matures with motor development (16), future research could investigate topographic working memory at the ages of seven or 8 years in children with motor disabilities, as well as after intervention measures. *Furthermore, since groups were well-defined with respect to topographical working memory using the WalCT test, visuospatial working memory using the CBT test and capability to reason on visuospatial material using the CPM test, this study may play a vital role in enhancing cognitive development among children with MMC and AMC*.

## Conclusion

Among the MMC and AMC groups, participants in the community and non-ambulation groups achieved similar topographic working memory scores, suggesting that persons using a wheelchair have the same possibility of participating actively in the environment as participants who commonly walk outdoors. This finding indicates that topographic working memory while walking is influenced to a similar extent as when being mobile in a wheelchair. The results may thus shed light on the importance of offering way-finding activities to children with low levels of MMC and AMC. Integrating aspects of navigation could thus be recommended during childhood as part of a therapy program, even for patients with low levels of MMC and AMC with community ambulation.

## Data Availability Statement

The raw data were generated at Karolinska Institutet. Derived data supporting the findings of this study are available from the corresponding author ÅB on request.

## Ethics Statement

The study was approved by the Regional Ethical Review Board in Stockholm. Parents gave informed written consent, and children provided verbal assent before taking part in the study.

## Author Contributions

ÅB: methodology, formal analysis, investigation, data curation, writing - original draft, writing - review, and editing. CG and LP: conceptualization, methodology, writing, review, and editing. All authors have read and approved the final version of the manuscript.

## Funding

This study was financially supported through grants from Promobilia Foundation Stockholm, Sweden.

## Conflict of Interest

The authors declare that the research was conducted in the absence of any commercial or financial relationships that could be construed as a potential conflict of interest.

## Publisher's Note

All claims expressed in this article are solely those of the authors and do not necessarily represent those of their affiliated organizations, or those of the publisher, the editors and the reviewers. Any product that may be evaluated in this article, or claim that may be made by its manufacturer, is not guaranteed or endorsed by the publisher.
